# Kidney-Sparing Methods for Extended-Field Intensity-Modulated Radiotherapy (EF-IMRT) in Cervical Carcinoma Treatment

**DOI:** 10.1371/journal.pone.0156623

**Published:** 2016-06-03

**Authors:** Hiroaki Kunogi, Nanae Yamaguchi, Yasuhisa Terao, Keisuke Sasai

**Affiliations:** 1 Department of Radiation Oncology, Juntendo University, 2-1-1, Hongo, Bunkyo-ku, Tokyo, 113–8421, Japan; 2 Department of Gynecology, Juntendo University, 2-1-1, Hongo, Bunkyo-ku, Tokyo, 113–8421, Japan; North Shore Long Island Jewish Health System, UNITED STATES

## Abstract

Coplanar extended-field intensity-modulated radiation therapy (EF-IMRT) targeting the whole-pelvic and para-aortic lymph nodes in patients with advanced cervical cancer results in impaired creatinine clearance. An improvement in renal function cannot be expected unless low-dose (approximately 10 Gy) kidney exposure is reduced. The dosimetric method should be considered during EF-IMRT planning to further reduce low-dose exposure to the kidneys. To assess the usefulness of non-coplanar EF-IMRT with kidney-avoiding beams to spare the kidneys during cervical carcinoma treatment in dosimetric analysis between non-coplanar and coplanar EF-IMRT, we compared the doses of the target organ and organs at risk, including the kidney, in 10 consecutive patients. To estimate the influence of EFRT on renal dysfunction, creatinine clearance values after treatment were also examined in 18 consecutive patients. Of these 18 patients, 10 patients who were included in the dosimetric analysis underwent extended field radiation therapy (EFRT) with concurrent chemotherapy, and eight patients underwent whole-pelvis radiation therapy with concurrent chemotherapy to treat cervical carcinoma between April 2012 and March 2015 at our institution. In the dosimetric analysis, non-coplanar EF-IMRT was effective at reducing low-dose (approximately 10 Gy) exposure to the kidneys, thus maintaining target coverage and sparing other organs at risk, such as the small bowel, rectum, and bladder, compared with coplanar EF-IMRT. Renal function in all 10 patients who underwent EFRT, including coplanar EF-IMRT (with kidney irradiation), was low after treatment, and differed significantly from that of the eight patients who underwent WPRT (no kidney irradiation) 6 months after the first day of treatment (*P* = 0.005). In conclusion, non-coplanar EF-IMRT should be considered in patients with advanced cervical cancer, particularly in patients with a long life expectancy or with pre-existing renal dysfunction.

## Introduction

Locally advanced cervical cancer often causes para-aortic lymph node metastases in 5% of stage I, 16% of stage II and 25% of stage III disease [1]. Extended-field radiation therapy (EFRT) targeting the whole-pelvic and para-aortic lymph nodes is an effective treatment option in patients with advanced cervical cancer [2]. Although para-aortic lymph node metastasis is defined as distant metastasis, cervical cancer patients with this metastasis type are often treated with EFRT and can be cured.

Prospective phase II cooperative group trials have reported 49% Grade 3 to 4 acute bowel toxicity with extended field chemo-radiotherapy using both 5-fluorouracil and cisplatin [3]. Intensity-modulated radiation therapy (IMRT) in the field of gynecological cancer has been shown to reduce bowel toxicity [4–7]. Beriwal et al. reported that EF-IMRT combined with concurrent single-agent chemotherapy was as effective as more aggressive treatment but with lower gastrointestinal toxicity. This result enhanced the possibility of EF-IMRT as a standard treatment option, considering the reduction of small bowel exposure and bowel toxicity.

The risk of renal dysfunction after RT and renal hypertension is well known, and the reduction in renal dose is intended in radiotherapy planning [8–11]. Regarding the influence of the applied dose to the kidneys, it is clear that a lower renal dose results in better renal function because 19% of patients who received a low dose (less than 12 Gy) showed impaired creatinine clearance [8]. Particularly, this should be considered in patients with long life expectations after curative treatment. The low-dose (approximately 10 Gy) constraint is important for the kidneys during kidney-sparing IMRT. In general, EF-IMRT planning goals provide the PTV prescribed dose coverage while minimizing the dose delivered to organs at risk, such as the small bowel. EF-IMRT planning may often be accomplished keeping the coverage of PTV with compromising kidney constraints because it is difficult to give kidney constraints the top priority, and the kidney constraint was relaxed depending on the PTV configuration and volume. Especially, in the context of coplanar EF-IMRT, it is difficult to reduce the radiation dose to the kidney when adequately irradiating the PTV. Varlotto et al. showed impaired creatinine clearance after coplanar EF-IMRT [12]. The normal kidney tissue constraint in their coplanar EF-IMRT plan was a maximum dose of 45 Gy with a maximum V16 of 35%. They reported that mean initial creatinine clearance decreased by 17.6% (from 109.23 mL/min before radiotherapy to 90.00 mL/min after radiotherapy). Thus, a dosimetric technique must be used to protect renal function and to further reduce radiation exposure. Needless to say, reducing the radiation field range for the related normal tissue volume in the IMRT plan reduces the related normal tissue volume irradiated with the lower dose. Non-coplanar EF-IMRT with kidney-avoiding beams reduces the renal volume irradiated with a low dose to a greater extent compared with that of coplanar EF-IMRT.

In EF-IMRT, the use of non-coplanar kidney-avoiding beams may reduce the kidney dose. If lower dose kidney sparing is accomplished easily using non-coplanar EF-IMRT, better results will be achieved with respect to late renal toxicity. The goal of this study was to evaluate the difference in low-dose kidney sparing for kidney protection using dose volume histogram (DVH) parameters between coplanar and non-coplanar EF-IMRT.

## Materials and Methods

### Patient characteristics

**Fig 1:**
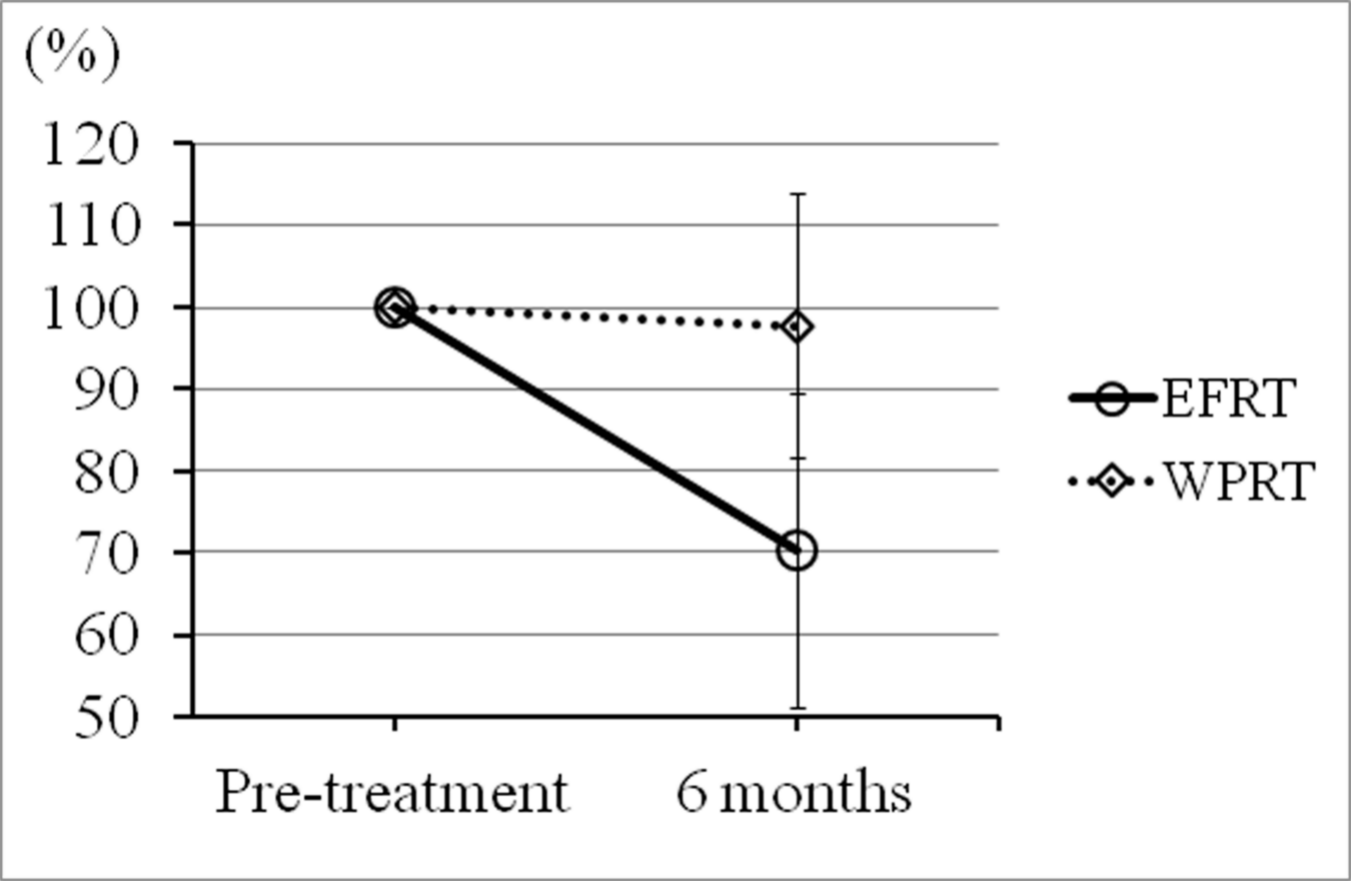
The ratios of pre-treatment creatinine clearance values to those 6 months after treatment (the pretreatment values were taken to be 100%). Mean ratios for all 10 EFRT patients and all 8 WPRT patients (○: EFRT and ◇: WPRT) are shown by the time elapsed from the first day of treatment. The renal function of the EFRT group (with kidney irradiation) was lower 6 months after treatment compared to those of the WPRT group (without kidney irradiation) (*P* = 0.005). Each error bar represents one standard deviation (SD) of the means for each group 6 months after treatment.

Eighteen consecutive patients underwent EFRT or whole-pelvis radiation therapy (WPRT) with concurrent chemotherapy (weekly cisplatin, 40 mg/m^2^) to treat cervical carcinoma between April 2012 and March 2015 at our institution. Both EFRT and WPRT were administered by reference to standard-of-care protocols. Written informed consent was obtained from all patients, who agreed to our use of their clinical data. This retrospective study was approved by the ethics committee of our institution (Juntendo University Hospital; approval no. 15–106) (S1 Fig), who waived the need for informed consent. Of these 18 patients, 10 who underwent EFRT were enrolled in the present non-interventional retrospective planning study. These 10 cervical cancer patients underwent EFRT (doses of 50.4 Gy in 28 fractions with a sequential additional boost for involved nodes) with concurrent use of chemotherapy (weekly cisplatin, 40 mg/m^2^). High-dose-rate (HDR) brachytherapy was performed using the tandem and ovoid applicator. After external beam therapy, three fractions, each of 6 Gy, were delivered to point A, with one fraction per week, in all 18 patients. However, these HDR brachytherapy doses were excluded from the dosimetric analysis, because they were not delivered to either kidney and thus did not affect the results and conclusions of the present study. Eligibility criteria for EFRT were para-aortic lymph node metastasis on positron emission tomography/computed tomography (PET/CT) or computed tomography (CT). Patient characteristics are given in Tables 1 and 2 refer to data on the EFRT and WPRT groups, respectively. To confirm the influence of EFRT on renal dysfunction in a clinical setting, creatinine clearance values at pre-treatment, and 6 months after the first day of treatment were examined, and the ratios of the creatinine clearance values 6 months after treatment to the pre-treatment values are shown in Fig 1. To explore the effect of concurrent chemotherapy (cisplatin, 40 mg/m^2^ weekly) on renal function, the creatinine clearance values pre-treatment, and at 6 months after treatment of the eight patients who underwent WPRT (without kidney irradiation) were examined. The ratios of pre-treatment to the later values are shown for reference in Fig 1.

**Table 1:** Patient characteristics (Extended-field radiation therapy).

Patient	Age	Histology	T	N	M	Planning	Chemotherapy	Pre-cre (mg/dl)^1^
1	44	SqCC^2^	1b1	1	1	coplanar IMRT	wCDDP40 mg/m^2(3)^	0.51
2	46	SqCC	2b	1	1	coplanar IMRT	wCDDP40 mg/m^2^	0.47
3	59	SqCC	3b	1	1	coplanar IMRT	wCDDP40 mg/m^2^	0.47
4	35	SqCC	2b	1	1	box field	none	0.45
5	38	SqCC	3b	1	1	box field	wCDDP40 mg/m^2^	0.37
6	61	SqCC	3b	1	1	box field	wCDDP40 mg/m^2^	0.55
7	54	SqCC	2b	1	1	box field	wCDDP40 mg/m^2^	0.47
8	57	SqCC	4a	1	1	box field	wCDDP40 mg/m^2^	0.52
9	43	SqCC	2b	1	1	box field	wCDDP40 mg/m^2^	0.42
10	38	SqCC	3b	1	1	box field	wCDDP40 mg/m^2^	0.58

**Table 2:** Patient characteristics (Whole-pelvis radiation therapy).

Patient	Age	Histology	T	N	M	Planning	Chemotherapy	Pre-cre (mg/dl)^1^
11	81	SqCC^2^	1b1	0	0	box field	none	0.48
12	73	SqCC	1b1	0	0	box field	none	0.54
13	61	SqCC	3b	1	0	box field	wCDDP40 mg/m^2(^^3^^)^	0.75
14	40	SqCC	4a	0	0	box field	wCDDP40 mg/m^2^	0.75
15	54	SqCC	2b	0	0	box field	wCDDP40 mg/m^2^	0.65
16	61	SqCC	2b	0	0	box field	wCDDP40 mg/m^2^	0.36
17	49	SqCC	2b	1	0	box field	wCDDP40 mg/m^2^	0.52
18	55	SqCC	2b	1	0	box field	wCDDP40 mg/m^2^	0.5

^1^Creatinine clearance values pre-treatment

^2^Squamous cell carcinoma

^3^Weekly cisplatin, 40 mg/m^2^.

### Radiation therapy structure delineation

For radiotherapy planning, CT was performed at slice thicknesses of 3 or 5 mm using a CT scanner (Hi-Speed Dxi; GE Healthcare, Buckinghamshire, UK) (Aquilion LB; TOSHIBA Medical Systems, Tochigi, JP). The clinical target volume (CTV) was contoured on the individual axial CT slices from each patient. The overall CTV included both the primary CTV and nodal CTV, including the pelvic and para-aortic lymph nodes. The pelvic lymph nodes were delineated on the planning CT in accordance with the Japan Clinical Oncology Group Gynecologic Cancer Study Group (JCOG-GCSG) consensus guidelines for the delineation of CTV for pelvic lymph nodes [13]. The CTV in the para-aortic region was contoured as the region between the psoas muscles, superiorly above the level of the renal artery (to the level of median T12/L1), and anteriorly encompassed the aorta and inferior vena cava with at least a 0.7-cm margin. The CTV was isotropically expanded by 7 mm to create the planning target volume (PTV). In addition, organs at risk (OARs), including the small bowel (contoured as a peritoneal space), rectum, bladder (both contoured as a whole organ), both kidneys, and spinal cord were delineated according to normal tissue contouring guidelines [14,15]. No margin was added to the contoured OAR.

### Comparison of treatment planning between coplanar and non-coplanar IMRT in dosimetric analysis

In terms of dosimetric analysis comparing coplanar and non-coplanar IMRT, only the EFRT dose of 50.4 Gy, thus excluding the sequential boost dose, was evaluated, because delivery of a sequential boost depended on the region of the involved node that was irradiated. Inverse treatment plans were generated for all coplanar or non-coplanar IMRT plans using seven fixed-fields of the Eclipse Planning System (version 11.0; Varian Medical Systems, Palo Alto, CA, USA). To avoid the bilateral kidneys, the gantry angles of most plans were 0°, 50°, 85°, 155°, 205°, 275°, and 310°. Non-coplanar EF-IMRT plans were generated using seven non-coplanar beams (combined with three coplanar anterior beams, two lateral inferior oblique beams and two posterior inferior oblique beams) and oblique beams designed to reduce irradiation of the kidney volume at the same gantry angles used in the coplanar plan. The two lateral inferior and two posterior inferior oblique beams were 85°/340°, 275°/20°, 155°/340°, and 205°/20° in terms of gantry/couch angles, respectively. All patients were treated with 10-MV photons.

EF-IMRT plans were optimized using the “Normal Tissue Objective” function of the Eclipse Planning System to generate optimal plans that spare the OARs (the small bowel, bladder, and rectum); the OAR constraints depended on the PTV configuration and volume. Other parameters considered during EF-IMRT planning were as follows: (top priority) 100% of the PTV receives 95% of the prescription dose, 95% of the PTV receives 100% of the prescription dose, and 0% of the PTV receives 110% of the prescription dose. Without decreasing the PTV coverage, we thereby maximally reduced kidney exposure by tightly reconfiguring the kidney constraints (at 20, 15, and 10 Gy). To avoid bias in the comparison between coplanar and non-coplanar IMRT plans, and also because the kidney optimization constraints of coplanar IMRT were looser than those of non-coplanar IMRT, we initially used the same kidney constraints (no more than 20% at 20 Gy, no more than 30% at 15 Gy, and no more than 45% at 10 Gy) in each plan (coplanar and non-coplanar). After that, the kidney constraints (at 20, 15, and 10 Gy) were gradually increased in severity without deteriorating the PTV coverage and with sparing of other OARs. As a result, for example, each kidney constraint was set at no more than 20% at 20 Gy, no more than 30% at 15 Gy, and no more than 45% at 10 Gy in the coplanar plan; and at no more than 10% at 20 Gy, no more than 15% at 15 Gy, and no more than 20% at 10 Gy in the non-coplanar plan. In each plan, we sought to (at least) meet the kidney constraint (no more than 35% at 16 Gy) of Gerszten et al. and Varlotto et al. [11, 12]. The dose distributions for both coplanar and non-coplanar EF-IMRT were optimized to achieve the lowest kidney dose while maintaining PTV coverage and sparing the OARs (the small bowel, bladder, and rectum); a plan was accepted if 95% of the PTV volume received 98% of the prescription dose, with the maximum dose being <110%, with 98% of the PTV receiving 95% of the prescription dose, and 50% of the PTV receiving 100% of that dose.

### Treatment plan evaluation between coplanar and non-coplanar IMRT in dosimetric analysis

To verify the validity of the PTV dose coverage and DVH parameters for OARs, we compared each V95 (i.e., the percentage of PTV covered by 95% of the prescription dose), each D95 and D50 (i.e., the minimum dose received by 95% and 50% of the PTV expressed as a percentage of the prescription dose, respectively), each D0.1cc (i.e., the minimum dose received by 0.1 cc of the PTV expressed as a percentage of the prescription dose), each RKV10, RKV15, RKV20, and RKV25 (i.e., the percent volumes of the right kidney receiving 10 Gy, 15 Gy, 20 Gy, and 25Gy, respectively), each LKV10, LKV15, LKV20, and LKV25 (i.e., the percent volumes of the left kidney receiving 10 Gy, 15 Gy, 20 Gy, and 25 Gy, respectively), each SV20, SV30, SV40, and SV50 (i.e., the percent volumes of the small bowel receiving 20 Gy, 30 Gy, 40 Gy, and 50 Gy, respectively), each RV30, RV40 and RV50 (i.e., the percent volumes of the rectum receiving 30 Gy, 40 Gy and 50 Gy, respectively), each BV30, BV40 and BV50 (i.e., the percent volumes of the bladder receiving 30 Gy, 40 Gy and 50 Gy, respectively), each RFV30, RFV40 and RFV50 (i.e., the percent volumes of the right femoral head receiving 30 Gy, 40 Gy and 50 Gy, respectively), each LFV30, LFV40 and LFV50 (i.e., the percent volumes of the left femoral head receiving 30 Gy, 40 Gy and 50 Gy, respectively), and CD0.1cc (i.e., the minimum dose received by 0.1 cc of the cord) between coplanar and non-coplanar EF-IMRT.

### Statistics

Differences in the mean value of each parameter were compared using Wilcoxon’s ranked sum non-parametric test (distribution-free method) appropriate when assessing the differences between coplanar EF-IMRT and non-coplanar EF-IMRT plans, because the number of samples was not large, and it was difficult to assume a normal distribution. Statistical analyses were performed using SPSS ver. 18 (SPSS Inc., Chicago, IL, USA). *P-*values less than 0.05 were considered to indicate statistical significance.

## Results

All 18 patients completed their courses of radical radiotherapy. The ratios of pre-treatment creatinine clearance values to those obtained 6 months after the first day of treatment are shown in Fig 1. The renal function of all 10 patients who underwent EFRT (with kidney irradiation), including coplanar EF-IMRT, was low after treatment (Fig 1). The renal function of all 10 patients who underwent EFRT (with kidney irradiation) differed significantly from those of the 8 patients who underwent WPRT (thus without kidney irradiation) 6 months after the first day of treatment (Fig 1; *P* = 0.005).

**Fig 2:**
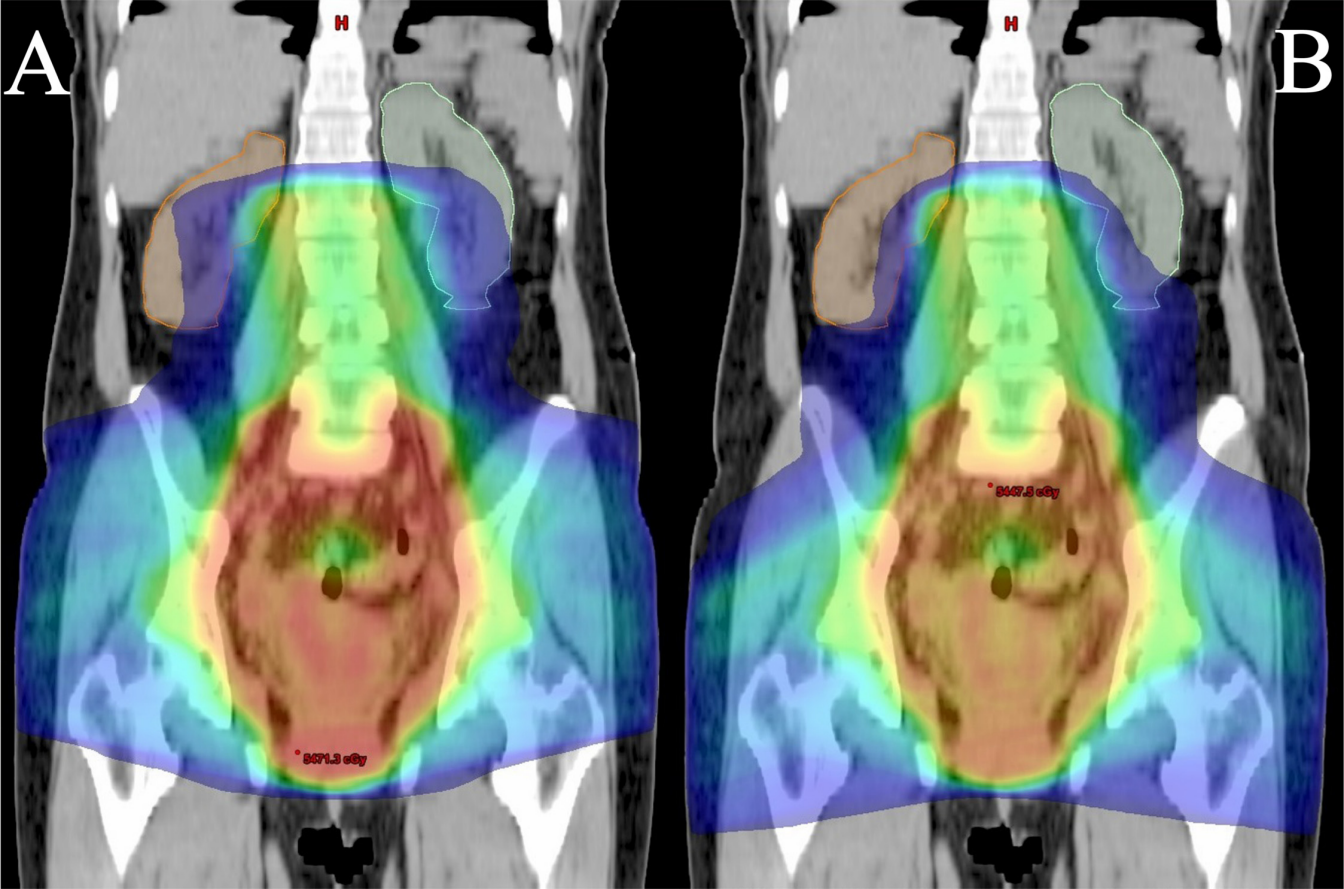
**Examples of coronal images and isodose distributions in the coplanar (A) and non-coplanar (B) EF-IMRT plans of a representative patient.** On both images, the extents of the right (brown line) and left (cyan line) kidney receiving the blue isodose color wash (10 Gy) are shown. A shift in the isodose distribution is apparent. The kidney area receiving the blue isodose color wash (10 Gy) on non-coplanar EF-IMRT is less than that on coplanar EF-IMRT.

The results of the analysis of dose-volume histograms (DVHs) were compared between coplanar and non-coplanar EF-IMRT for the 10 cases who underwent EFRT (Table 3). The mean PTV V95, D95, D50 and D0.1cc (as percentages) for the 10 cases were 99.2%, 98.7%, 104.0%, and 109.0 for coplanar EF-IMRT and 99.1%, 98.7%, 104.0%, and 109.0% for non-coplanar EF-IMRT, respectively. There were no significant differences. RKV10, RKV15, RKV20, LKV10, LKV15, and LKV20 were significantly reduced from means of 56.4%, 29.2%, 14.4%, 55.8%, 30.7%, and 15.7%, respectively, in the coplanar plans to means of 39.8%, 18.5%, 9.2%, 37.8%, 19.8%, and 9.8%, respectively, in the non-coplanar plans (Table 3). There were no significant differences between coplanar and non-coplanar EF-IMRT in the mean values of RKV25, LKV25, SV20, SV30, SV40, SV50, RV30, RV40, RV50, BV30, BV40, BV50, RFV30, RFV40, RFV50, LFV30, LFV40, LFV50, and CD0.1cc (Table 3). Fig 2 illustrates coronal images of the right and left kidney receiving 10 Gy when the coplanar and non-coplanar EF-IMRT techniques were used on a representative patient, respectively.

**Table 3:** Comparisons of the dose-volume histograms (DVHs) of coplanar and non-coplanar EF-IMRT.

	Coplanar		Non-coplanar		Mean difference^1^	P value
	Mean	Range	Mean	Range		
**PTV**						
V95 (%)^2^	99.2	98.9–99.3	99.1	98.8–99.3	0.1	0.24
D95 (%)^3^	98.7	98.2–99.2	98.7	98.0–99.0	0	0.97
D50 (%)^3^	104	103.6–104.6	104	103.2–105.0	0	0.85
D0.1cc (%)^4^	109	108.7–109.1	109	108.8–109.0	0	0.3
**Right kidney**						
RKV10 (%)^5^	56.4	50.8–61.8	39.8	30.8–54.1	16.6	< 0.001
RKV15 (%)^5^	29.2	20.1–37.2	18.5	11.0–31.2	10.7	0.003
RKV20 (%)^5^	14.4	7.6–23.7	9.2	2.7–20.3	5.2	0.038
RKV25 (%)^5^	5.4	0.5–14.9	3.7	0.0–13.9	1.7	0.19
**Left kidney**						
LKV10 (%)^6^	55.8	45.5–63.1	37.8	26.5–49.9	18	< 0.001
LKV15 (%)^6^	30.7	21.3–37.4	19.8	11.0–26.2	10.9	< 0.001
LKV20 (%)^6^	15.7	8.3–21.2	9.8	4.0–15.4	5.9	0.007
LKV25 (%)^6^	5.2	1.7–10.0	3.5	0.5–8.1	1.7	0.12
**Small bowel**						
SV20 (%)^7^	76.8	64.6–92.9	73.9	63.2–91.3	2.9	0.38
SV30 (%)^7^	47.6	30.0–63.9	49.3	31.6–69.1	-1.7	0.62
SV40 (%)^7^	28.8	14.5–42.3	29.3	15.1–43.3	-0.5	0.73
SV50 (%)^7^	17.3	7.3–27.4	17.3	6.8–27.6	0	1
**Rectum**						
RV30 (%)^8^	98.6	93.6–100	99	94.7–100	-0.4	0.45
RV40 (%)^8^	97.1	91.7–100	97.3	92.5–100	-0.2	0.85
RV50 (%)^8^	91.5	87.4–97.9	91.3	86.6–97.4	0.2	0.73
**Bladder**						
BV30 (%)^9^	100	99.9–100	100	99.9–100	0	1
BV40 (%)^9^	94.4	78.4–100	97.2	91.2–100	-2.8	0.57
BV50 (%)^9^	69.7	34.9–98.5	68.7	31.2–97.3	1	0.85
**Right femoral head**						
RFV30 (%)^10^	36.3	0–95.7	35.5	27.6–38.9	0.8	0.21
RFV40 (%)^10^	3.7	0–1.4	5.1	0.1–9.9	-1.4	0.38
RFV50 (%)^10^	0	0–0	0	0–0	0	-
**Left femoral head**						
LFV30 (%)^11^	36.9	26.3–78.3	35	29.0–47.2	1.9	0.43
LFV40 (%)^11^	6.6	0–33.9	4.5	0–17.4	2.1	0.88
LFV50 (%)^11^	0.7	0–6.6	0	0–0	0.7	0.37
**Cord**						
CD0.1cc (Gy) ^12^	40.4	37.5–43.8	42.3	39.5–44.3	-1.9	0.07

^1^Comparisons between coplanar and non-coplanar plans

^2^V95 –the percentage of the PTV covered by 95% of the prescription dose

^3^D95 and D50 –the minimum doses received by 95% and 50% of the PTV, expressed as percentages of the prescription dose, respectively

^4^D0.1cc–the minimum dose received by 0.1 cc of the PTV expressed as a percentage of the prescription dose

^5^RKV10, RKV15, RKV20, and RKV25 –the percentage volumes of the right kidney receiving 10, 15, 20, and 25 Gy, respectively

^6^LKV10, LKV15, LKV20, and LKV25 –the percentage volumes of the left kidney receiving 10, 15, 20, and 25 Gy, respectively

^7^SV20, SV30, SV40, and SV50 –the percentage volumes of the small bowel receiving 20, 30, 40, and 50 Gy, respectively

^8^RV30, RV40, and RV50 –the percentage volumes of the rectum receiving 30, 40, and 50 Gy, respectively

^9^BV30, BV40, and BV50 –the percentage volumes of the bladder receiving 30, 40, and 50 Gy, respectively

^10^RFV30, RFV40, and RFV50 –the percentage volumes of the right femoral head receiving 30, 40, and 50 Gy, respectively

^11^LFV30, LFV40, and LFV50 –the percentage volumes of the left femoral head receiving 30, 40, and 50 Gy, respectively

^12^CD0.1cc–the minimum dose received by 0.1 cc of the cord.

## Discussion

This is the first study to evaluate the dosimetric utility of kidney-sparing non-coplanar EF-IMRT for cervical cancer treatment. Coplanar EF-IMRT targeting whole-pelvic and para-aortic lymph nodes in patients with advanced cervical cancer results in impaired creatinine clearance [12]. An improvement in renal function is difficult unless the low-dose (approximately 10 Gy) exposure kidney volume is reduced during EF-IMRT planning. A dosimetric technique must be used during EF-IMRT planning to further reduce kidney radiation exposure. Non-coplanar EF-IMRT with kidney-avoiding beams reduces the renal volume irradiated with a low dose to a greater extent, and we therefore suggest kidney-sparing non-coplanar EF-IMRT as a standard treatment method for EF-IMRT planning. Such kidney-sparing methods for EF-IMRT of cervical cancer will provide a rationale for subsequent clinical studies.

EFRT including EF-IMRT will be a standard treatment option for advanced cervical cancer patients with para-aortic metastatic lymph nodes in the future. EF-IMRT can improve with respect to small bowel-sparing compared with conventional EFRT (box field). EF-IMRT often delivers the radiation dose using coplanar beams to avoid the surrounding normal tissues [11,12,16,17]. This dosimetric study demonstrated the feasibility of generating clinically acceptable and deliverable kidney-sparing non-coplanar EF-IMRT plans for cervical cancer patients who need whole-pelvic and para-aortic radiation therapy. If kidney sparing is intended using non-coplanar beams, better kidney protection will be accomplished. Indeed, the creatinine clearance values of all 10 patients who underwent EFRT, including coplanar EF-IMRT, were impaired after treatment. The renal function of patients who underwent EFRT (with kidney irradiation) differed significantly from those of patients who underwent WPRT (thus without kidney irradiation) (Fig 1).

To reduce low-dose exposure to the kidney, the incident beam directions through the kidney must be adjusted to reduce the irradiated kidney area. In this study, the CTV is contoured up to the region superiorly above the level of the renal artery, so that each kidney is located near the PTV and tends to be irradiated with a few incident beams. In coplanar EF-IMRT planning, there is a limit on the reduction of low-dose exposure to the kidney located near the PTV, while keeping the coverage of the PTV and sparing other organs at risk such as the small bowel. In this study, in non-coplanar EF-IMRT planning, we applied the same beam angles as in each coplanar plan to prevent the coverage of the PTV and OAR sparing from being made considerably different by using different beam angles. The four incident coplanar beams on the obliquely rear side passing through the edge of each kidney were changed into four non-coplanar beams on the obliquely lower rear side (two lateral inferior oblique beams and two posterior inferior oblique beams). The couch on the obliquely rearward coplanar beam was rotated, and the rotation angle was set to 20°, preventing interference between the gantry and couch. Needless to say, in IMRT planning, the reduction in the radiation field range for the related normal tissue volume results in the reduction of the related normal tissue volume irradiated with a lower dose. Non-coplanar EF-IMRT with kidney-avoiding beams can more largely reduce the renal volume irradiated with a low dose compared with coplanar EF-IMRT. Aimed at protecting the kidneys, the kidney-sparing method using non-coplanar EF-IMRT for advanced cervical cancer patients can be performed easily in the clinical setting at any institution.

The volumes of each kidney with irradiation dose ranges of 10 to 20 Gy in non-coplanar EF-IMRT were significantly lower than those in coplanar EF-IMRT (Table 3). There were no significant differences in PTV, small bowel, rectum, bladder, femoral head, and cord doses between coplanar and non-coplanar EF-IMRT (Table 3). These results show that non-coplanar planning maintains the PTV coverage and OAR constraints even if non-coplanar beams are used. Lower doses to the kidney were effectively decreased by non-coplanar EF-IMRT keeping the coverage of the PTV and other organs at risk. Non-coplanar EF-IMRT may be necessary in patients with pre-existing renal dysfunction.

In 3D conformal radiotherapy, Lawrence et al. showed that no more than 55% of the volume of the bilateral whole kidney should receive a dose greater than 12 Gy [9]. Coplanar EF-IMRT was limited by reducing the volume of the kidney to receive a low dose such as ~10 Gy, keeping the coverage of PTV and avoiding damage to other organs at risk. Gerszten et al. showed that kidney constraints were no more than 35% at 16 Gy using EF-IMRT with seven coplanar beams, which was prescribed at 45 Gy in 25 fractions for the PTV [11]. Varlotto et al. also showed impaired creatinine clearance after coplanar EF-IMRT [12]. The normal kidney tissue constraint in their coplanar EF-IMRT plan was a maximum dose of 45 Gy with a maximum V16 of 35%; mean V15/V20 values for the kidneys and median kidney dose were 25.41 mL/13.37 mL and 10.78 Gy, respectively. These parameters are similar to those used in our coplanar EF-IMRT plan. The previous study reported that mean initial creatinine clearance decreased 17.6% (from 109.23 mL/min before radiotherapy to 90.00 mL/min after radiotherapy). Even if kidney sparing was intended, lower dose kidney protection by coplanar EF-IMRT might often be difficult. However, non-coplanar EF-IMRT accomplished substantial kidney sparing such that each median kidney was spared (38.8% at 10 Gy, 19.2% at 15 Gy, and 9.5% at 20 Gy).

The non-coplanar technique of radiation therapy may deliver the radiation dose while avoiding surrounding normal tissues, and its advantage has been proven in other anatomical sites such as head and neck cancer [18,19]. The non-coplanar IMRT plan using oblique beams to avoid the kidney has an advantage of delivering the radiation dose while avoiding not only surrounding normal tissues in the pelvic site, but also the kidney in the abdominal site.

In non-coplanar EF-IMRT, there may be a larger influence on the setup error accounting for patient motion and setup uncertainty compared with coplanar EF-IMRT, even if the angles from the horizontal plane on non-coplanar beams are no more than 20 degrees. A non-coplanar EF-IMRT plan will need a more accurate setup with great care.

In a comparison between coplanar and non-coplanar IMRT plans, it may be difficult to avoid bias in each plan completely. In this study, we used the same constraints for both PTV and OARs in each plan and then tightly reconfigured the kidney constraints without adversely affecting PTV coverage, avoiding a biased comparison in that the kidney optimization constraints of coplanar IMRT were looser than those of non-coplanar IMRT. Even if it is difficult to avoid bias completely, non-coplanar plans that adjust the incident beam directions through the kidney and reduce the irradiated kidney area have merit in reducing low-dose exposure to the kidney.

One limitation of the present study was that the extent of kidney irradiation from EF-IMRT for cervical cancer is dependent on the anatomical location of each kidney with respect to the PTV position. The positional relationship between the right and left kidney is also an important factor. The positional relationship between the PTV and the kidney can influence the direction of the non-coplanar beams to avoid the bilateral kidneys. In the case that one kidney is located at a position considerably lower than the other kidney, even if the non-coplanar technique is used, it will be difficult to reduce the lower dose to the kidney lying at a lower position.

This study focused on generating kidney-sparing EF-IMRT plans using non-coplanar beams. The potential sparing benefits of non-coplanar EF-IMRT for other OARs, such as bone marrow [20], will be investigated in the future.

## Conclusions

Non-coplanar EF-IMRT was effective at reducing low-dose (approximately 10 Gy) exposure to the kidneys without compromising PTV coverage and other organs at risk, and should be considered in patients with advanced cervical cancer, particularly those with a long life expectancy or those with pre-existing renal dysfunction.

## Supporting Information

S1 Fig Our ethical approval document. This retrospective study was approved by the ethics committee of our institution (Juntendo University Hospital; approval no. 15–106).
(PDF)

## Abbreviations

CT: computed tomography
CTV: clinical target volume
DVH: dose-volume histogram
D0.1cc: minimum dose received by 0.1 cc of the PTV volume expressed as a percentage of the prescription dose
D95 and D50: minimum dose received by 95% and 50% of the PTV volume expressed as a percentage of the prescribed dose, respectively
EF-IMRT: extended-field intensity-modulated radiation therapy
EFRT: extended-field radiation therapy
HDR: high-dose-rate
IMRT: intensity-modulated radiation therapy
JCOG-GCSG: Japan Clinical Oncology Group Gynecologic Cancer Study Group
OAR: organs at risk
PET/CT: positron emission tomography/computed tomography
PTV: planning target volume
V95: percentage of PTV volume covered by 95% of the prescribed dose
WPRT: whole-pelvis radiation therapy
