# Supplementary material for: Kidney-Sparing Methods for Extended-Field Intensity-Modulated Radiotherapy (EF-IMRT) in Cervical Carcinoma Treatment
Source: PLoS One. 2016 Jun 3;11(6):e0156623. doi: 10.1371/journal.pone.0156623 (PMC4892687; doi:10.1371/journal.pone.0156623)
Supplement: S1 Fig — This retrospective study was approved by the ethics committee of our institution (Juntendo University Hospital; approval no. 15–106). (PDF) [file pone.0156623.s001.pdf]

Juntendo, Tokyo, founded in 1838

Head Office
Juntendo University
Faculties & Graduate Schools
Hongo 2-1-1, Bunkyo-ku
Tokyo 113-8421, Japan

Juntendo University
Faculty of Medicine &
Graduate School of Medicine
Hongo 2-1-1, Bunkyo-ku
Tokyo 113-8421, Japan

Juntendo University
Faculty of Health and Sports Science &
Graduate School of Health and Sports Science
Hiraga-gakuendai 1-1, Inzai City
Chiba 270-1695, Japan

Juntendo University
Faculty of Health Care and Nursing &
Graduate School of Health Care and Nursing
Takasu 2-5-1, Urayasu City
Chiba 279-0023, Japan

Juntendo University
Faculty of Health Sciences and Nursing
Omiya-cho 3-7-33, Mishima City
Shizuoka 411-8787, Japan

Hospital Ethics Committee
Juntendo University Hospital

Date Oct/2/2015

Number of Research: 15-106
Title of Research:
Kidney-Sparing Methods for Extended-Field Intensity-Modulated Radiotherapy (EF-IMRT) in Cervical Carcinoma Treatment

Name(s) of Reseacher(s):
Hiroaki Kunogi
Nanae Yamaguchi
Yasuhisa Terao
Keisuke Sasai

This is to certify that the above mentioned research has been reviewed and approved by the committee.

Hiroyuki Daida ,M.D.,Ph.D
Director
Juntendo University Hospital
Hongo 3-1-3, Bunkyo-Ku
Tokyo, 113-8431, Japan
